# Estimating Acrylamide and 5-Hydroxymethylfurfural Levels in Crackers Using Computer Vision: Effects on Consumer Acceptance

**DOI:** 10.3390/foods15112011

**Published:** 2026-06-04

**Authors:** Franco Pedreschi, Darwin Castillo, Andrea Bunger, Romina Pedreschi, Diego García-Ríos, Juan E. Alvaro, María Salomé Mariotti-Celis, Marcela Medel-Maraboli, Américo Contreras, Domingo Mery

**Affiliations:** 1 Departamento de Ingeniería Química y Bioprocesos, Facultad de Ingeniería, Pontificia Universidad Católica de Chile, P.O. Box 306, Santiago 6904411, Chile; 2 Departamento de Ciencia de los Alimentos y Tecnología Química, Facultad de Ciencias Químicas y Farmacéuticas, Universidad de Chile, P.O. Box 233, Santiago 8380000, Chile; darwinrcb@gmail.com (D.C.);; 3 Escuela de Agronomía, Facultad de Ciencias Agronómicas y de los Alimentos, Pontificia Universidad Católica de Valparaíso, Calle San Francisco s/n, La Palma, Quillota 2260000, Chile; romina.pedreschi@pucv.cl (R.P.); diego.garcia.r@pucv.cl (D.G.-R.); juan-eugenio.alvaro@pucv.cl (J.E.A.); 4 Millennium Institute Center for Genome Regulation, Santiago 8320000, Chile; 5 Escuela de Nutrición y Dietética, Facultad de Medicina, Universidad Finis Terrae, Pedro de Valdivia 1509, Santiago 7501015, Chile; mmariotti@uft.cl; 6 Facultad de Ciencias Agronómicas, Universidad de Chile, Santa Rosa 11315, La Pintana, Santiago 8820808, Chile; mmedel@uchile.cl (M.M.-M.); a.contreras@uchile.cl (A.C.); 7 Departamento de Ciencia de la Computación, Facultad de Ingeniería, Pontificia Universidad Católica de Chile, P.O. Box 306, Santiago 6904411, Chile

**Keywords:** image analysis, cross-validation, GC-MS, HPLC-DAD, modeling, sensory testing

## Abstract

Crackers are a popular and convenient snack; however, the baking process can produce neo-formed contaminants (NFCs), such as acrylamide (AA) and 5-hydroxymethylfurfural (HMF), through non-enzymatic browning reactions. Conventional analytical methods for quantifying these NFCs are complex, labor-intensive, and require specialized personnel. The main objective of this study was to develop computer vision (CV) models based on surface digital image analysis for the rapid prediction of AA and HMF in crackers. Therefore, five baking temperatures (160, 170, 180, 190, and 200 °C) and times (15, 20, 25, 30, and 35 min) were tested and analyzed using CV alongside conventional analytical methods. CV estimates and analytical measurements for AA (4.35–829 µg kg^−1^) and HMF (0.004–105.4 mg kg^−1^) contents were compared using cross-validation with a “leave-one-treatment-out” approach. The average error for missing measurements was 3.10% for AA and 3.28% for HMF, validating CV as an efficient tool for the rapid estimation of these NFCs in crackers. Among the cracker samples with AA content below the EU benchmark of 400 μg/kg, evaluated using the Check-All-That-Apply (CATA) test, consumers preferred the samples baked at 180 °C for 25 min, which also exhibited the lowest levels of both AA and HMF.

## 1. Introduction

Bakery products are staple foods globally, supplying proteins, carbohydrates, fiber, and micronutrients [1]. Among them, crackers which are thin, dry baked products made primarily from soft wheat flour, are widely consumed (5.6 kg per capita in 2023, https://www.marketresearch.com/GlobalIndustry-Analysts-v1039/Crackers-0824702/ accessed on 20 March 2026) due to their affordability, convenience, and long shelf life [2,3,4].

However, high-temperature processing promotes the formation of neo-formed contaminants (NFCs), notably acrylamide (AA) and 5-hydroxymethylfurfural (HMF), mainly via the Maillard reaction (MR) and sugar degradation pathways [1,5,6,7]. AA, classified by the International Agency for Research on Cancer as a Group 2A probable carcinogen, is primarily generated through reactions between free asparagine and carbonyl compounds, with asparagine acting as a rate-limiting precursor [8,9,10]. Thus, the European Commission has established a benchmark level of 400 μg kg^−1^ for crackers [11]. In addition, HMF, formed through MR and sugar dehydration, is also associated with potential genotoxic, hepatotoxic, and carcinogenic effects, particularly following conversion to reactive intermediates [12,13,14,15,16,17,18,19].

A positive correlation between AA and HMF in thermally processed starchy foods suggests that HMF can serve as a practical proxy for AA due to its lower analytical cost and simpler quantification [20,21]. Conventional analytical techniques such as LC/GC-MS (Liquid chromatography/gas chromatography-mass spectrometry) and HPLC-DAD (High performance liquid chromatography-diode array detector) provide accurate quantification but are labor-intensive, costly, and unsuitable for real-time industrial applications [21,22,23,24,25].

Consequently, computer vision (CV) has emerged as a non-destructive, rapid, and cost-effective alternative for monitoring food quality through surface color and texture analysis [26,27,28]. Recent studies demonstrate high predictive performance of CV-based models for AA in potato chips, including hyperspectral imaging, convolutional neural networks, and machine learning approaches, achieving accuracies above 94–98% [29,30,31,32,33,34]. Similar approaches have been extended to other matrices such as toasted bread, with robust predictive capacity based on color and thermal features [35,36]. Nevertheless, the relationship between browning and AA is not always linear, limiting predictive robustness across heterogeneous food systems [27,29].

In parallel, sensory characterization remains critical in product development. Consumer-based methodologies such as the Check-All-That-Apply (CATA) test provide rapid and reliable sensory profiling by capturing consumer perceptions using predefined descriptors, typically requiring 50–100 participants [37,38,39,40,41].

While previous CV-based studies have successfully predicted AA utilizing deep learning or hyperspectral imaging, they often face implementation barriers due to high computational and data requirements. This study introduces a highly efficient, low-complexity methodology utilizing rotation-invariant Local Binary Patterns (LBPs) [42] to facilitate real-time industrial deployment. Furthermore, this study presents a novel comparative framework that tests the predictive limits of CV against chemical proxies (HMF), ultimately integrating these safety metrics with Check-All-That-Apply (CATA) sensory profiles to optimize both consumer acceptance and chemical safety.

## 2. Materials and Methods

### 2.1. Cracker Production

The cracker dough was prepared according to the formulation specified in Table 1, using commercial wheat flour without baking powder (Selecta brand, Santiago, Chile), vegetable lard (Astra brand, Chile), table salt (Lobos brand, Iquique, Chile), and warm water at 37 °C. These ingredients were mixed for 4 min until a homogeneous dough was achieved using a mixer machine (Pro Line^®^ Series 7 Quart Bowl-Lift Stand Mixer, KitchenAid, Troy, OH, USA), without any fermentation period. Subsequently, the dough was rolled out to a thickness of approximately 1 mm using an electric laminator (RAM, model Q50B/700, Schio, Italy), and circular pieces (4 cm in diameter) were cut using a stainless steel circular mold. The formed dough pieces were perforated at several points on their surface and then baked in a convection electric oven (Garbin Industries, model 43 DX UMI, Vicenza, Italy). Sixteen dough pieces were placed on each tray of the oven for each predetermined experimental baking condition as shown in Table 2, with temperatures ranging from 160 °C to 200 °C and durations spanning from 15 to 35 min. To ensure consistent heating, the convection oven was preheated and stabilized for 1 h. Finally, the crackers obtained under each baking condition were either allowed to cool at room temperature (20 °C) for immediate top surface image acquisition and sensory evaluation (when applicable) or stored in airtight low-density polyethylene bags and frozen at −20 °C for rapid subsequent AA and HMF analysis.

### 2.2. Analytical Determinations

#### 2.2.1. Acrylamide Analysis

The AA content in crackers was quantified using gas chromatography-mass spectrometry (GC-MS) based on the methodology of Ciesarová et al. [43], with some modifications. A two gram portion of homogenized cracker sample was placed in a centrifuge tube, followed by the addition of 40 μL of the internal standard (IS, AA-D3) working solution (20 mg/L) and 10 mL of methanol. The mixture was vortexed for 30 s and sonicated at 60 °C for 20 min. The samples were centrifuged at −4 °C at 6000 rpm. Five mL of the supernatant was collected and passed through a C18 reversed-phase cartridge, followed by an additional 5 mL of methanol. The extract was transferred to a 50 mL centrifuge tube, and 10 mL of n-hexane was added. The mixture was sonicated for 5 min and centrifuged at −10 °C at 6000 rpm for 10 min. The methanol phase (lower phase) was collected, and the solvent was evaporated at 40 °C until dry. One mL of methanol was added, and the solution was filtered through a 0.2 μm filter to reconstitute the extract; the final extract was placed in 2 mL vials for analysis. The analysis used an Agilent 7890A GC System equipped with an Agilent DB-FFAP column (0.25 µm, 30 m × 0.25 mm) and an Agilent 597C XL EI/CI MSD mass spectrometer (Agilent Technologies, Santa Clara, CA, USA). An injection volume of 2 µL in splitless-pulsed mode was used with a GC inlet temperature of 200 °C. The oven temperature gradient was as follows: started at 60 °C for 1 min, then increased to 190 °C at 10 °C min^−1^, then increased to 240 °C at 50 °C min^−1^. High purity helium was used as carrier gas at 0.8 mL min^−1^. The MS source and quadrupole temperatures were set at 150 °C, while the transfer line temperature was 250 °C. Data were acquired using SIM mode in negative polarity and AA (*m*/*z* = 70) and AA-D3 (*m*/*z* = 73) areas were measured. A standard curve was constructed using AA and AA-D3, and the ratios were calculated and used to calculate AA concentrations in the samples. The results were reported as µg of AA per kg of dry solids. The method validation parameters (linearity, precision and accuracy are shown in Supplementary Section S1.1.

#### 2.2.2. 5-Hydroxymethylfurfural Analysis

The HMF content was carried out by high-performance liquid chromatography coupled to a diode array detector (HPLC-DAD) according to the method reported by Toker et al. [44] with some modifications [45]. One gram of homogenized cracker sample was combined with 20 mL of distilled water, 1.5 mL of Carrez I, and 1.5 mL of Carrez II. The mixture was centrifuged at 4800 g for 15 min. The supernatant was collected and passed through a 0.22 μm syringe filter before being injected. Chromatographic separation was performed on an HPLC system with a diode array detector (Dionex UltiMate 3000, Thermo Scientific, Waltham, MA, USA) and an Acclaim TM 120 C18 column. The mobile phase comprised a 95% acetic acid solution (1% in water) and 5% acetonitrile (*v*/*v*). Isocratic elution was performed at a flow rate of 1 mL/min, with an injection volume of 20 μL and a detection wavelength of 284 nm. The results were expressed as mg of HMF per kg of dry solids. The method validation parameters (linearity, precision and accuracy are shown in Supplementary Section S1.2.

### 2.3. Statistical Design

A full factorial experimental design, comprising 25 combinations of independent heating variables for baking (5 temperatures and 5 times), was conducted with three replicates to investigate the effect of baking conditions (temperature: 160, 170, 180, 190, and 200 °C; time: 15, 20, 25, 30, 35 min) on the formation of AA and HMF in crackers, as outlined in Table 2.

Sixteen crackers were baked for each condition in triplicate, resulting in 48 samples per treatment, and photographs were taken of the top surface of each piece, totaling 1200 photographs. Simultaneously, for the laboratory experimental analysis of AA and HMF, samples from the three replicates for each baking condition were crushed and homogenized in one sample which was measured by duplicate.

### 2.4. Computer Vision Strategy

This section introduces the proposed computer vision strategy to estimate the formation of AA and HMF in crackers processed during baking at different high constant temperatures and different times. Our method consists of nine steps, each of which will be explained in further detail.

#### 2.4.1. Image Acquisition

**Figure 1:**
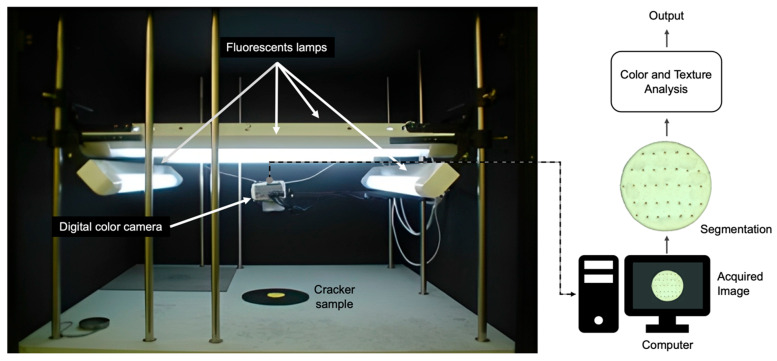
Computer vision system for the analysis of crackers.

The images of the cracker samples used for the experiment were meticulously acquired using a state-of-the-art laboratory image acquisition system (DVS-Computer Vision System, Divisol, Santiago, Chile), implemented by Pedreschi et al. [46], with some modifications as illustrated in Figure 1. This system consists of four fluorescent lamps (Halux, 18 W, 60 cm long) with a color temperature of 6500 K and a color rendering index (Ra) close to 95%. These lamps were arranged as a square 35 cm above the sample and at a 45° angle to the sample plane, ensuring a uniform light intensity to the sample. The D5600 digital color camera (DCC) (Nikon, Japan) was placed vertically at 22.5 cm from the sample at an angle of about 45° between the axis of the camera lens and the light sources. The lamps and the DCC were placed inside a wooden box with black inner walls to avoid light and reflection from the camera walls.

**Figure 2:**
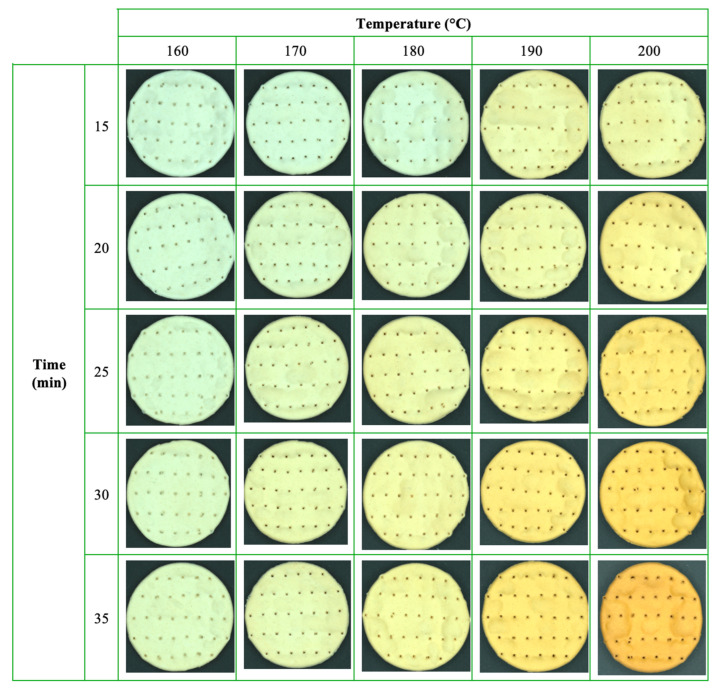
Surface top images of crackers from 25 baking treatments (5 different temperatures across 5 different times).

Images were captured with the DCC mentioned above manually set to its maximum resolution: 6016 × 4016 pixels; ISO speed: ISO-100, F-stop: f/5.6, exposure time: 1/60 s, flash mode: no flash, focal length: 55 mm, and connected to an ITR-N3 remote trigger (Godox, Shenzhen, China) via the N3 port of the camera. The acquired images, in original format NEF (Nikon Electronic Format, files that contain a RAW format unique to digital photographs taken with Nikon cameras). The raw image format contains the entire image data as captured by the camera’s digital sensor, photographic or otherwise. They were stored directly on the digital camera’s removable memory (PNY brand, 64 GB). In total, 25 × 16 × 3 = 1200 digital images (25 treatments, 16 crackers per treatment, and 3 replicates) were taken from the top side of the crackers, one at a time, under the above conditions. Top surface image examples of the 25 treatments are shown in Figure 2.

#### 2.4.2. Image Conversion

The original NEF images were converted to PNG format using Adobe’s Lightroom software (version 2023), a widely used tool for professional image editing. The images were then resized to 600 × 400 pixels using Matlab’s Image Processing Toolbox (version 2023), using a bicubic interpolation.

In our experiments, the 1200 color images were stored as I*_i_*, with

(1) $$i=48\left(t-1\right)+3\left(c-1\right)+r$$

where the indices are defined as follows:

*i* = 1, …, 1200: for the 1200 images.

*t* = 1, …, 25: for the 25 treatments,

*c* = 1, …, 16: for the 16 crackers per treatment, and

*r* = 1, …, 3: for the 3 replicates.

#### 2.4.3. Segmentation

Each color image was segmented using an algorithm implemented in Matlab (version 2022) and the Balu library [47]:

(2) $$J_{i}=segment\left(I_{i}\right)\;\mathrm{for}i=1,\;\ldots ,\;1200$$

where *J_i_* corresponds to the color pixels of the cracker of image *I_i_*.

#### 2.4.4. Feature Extraction

The rotation-invariant Local Binary Pattern (LBP) features [42] were extracted in the segmented color image only (the background of the cracker’s image is removed). Local Binary Pattern (LBP) features might be more practical than deep learning methods for some computer vision tasks because of their low computational complexity. LBP can be implemented and run efficiently even on hardware with limited processing power and memory. This simplicity also means that LBP requires significantly less data to perform effectively, avoiding the extensive data collection and training periods needed for deep learning models. Using the rotation-invariant LBP for texture features in color images of foods offers several advantages. This method is computationally efficient and straightforward, making it ideal for many applications. It is robust against changes in illumination and insensitive to rotations, capturing consistent textures across various orientations of food items, such as the surface textures or the smoothness of the crackers. When applied to individual color channels, it effectively extracts color texture features, which is crucial for assessing the variations in each color channel. Additionally, the rotation-invariant LBP can be easily integrated with the next steps of our proposed method. In our case we use the rotation-invariant version of Balu library [47] in the R, G, and B channels of each segmented image giving 36 features per color channel. In this way, for each image, an LBP descriptor of 36 × 3 = 108 elements is obtained. Thus, for the 1200 color images, we store this information in a 1200 × 108 element matrix, called X. In this case, the *i*-th row of the matrix is:

(3) $$x_{i}={LBP}_{ri}\left(J_{i}\right)\;\mathrm{for}i=1,\;\ldots ,\;1200$$

where *x_i_* corresponds to the 108 LBP rotation-invariant features of the color pixels of image *I_i_*, here we use the corresponding segmentation image *J_i_*.

**Figure 3:**
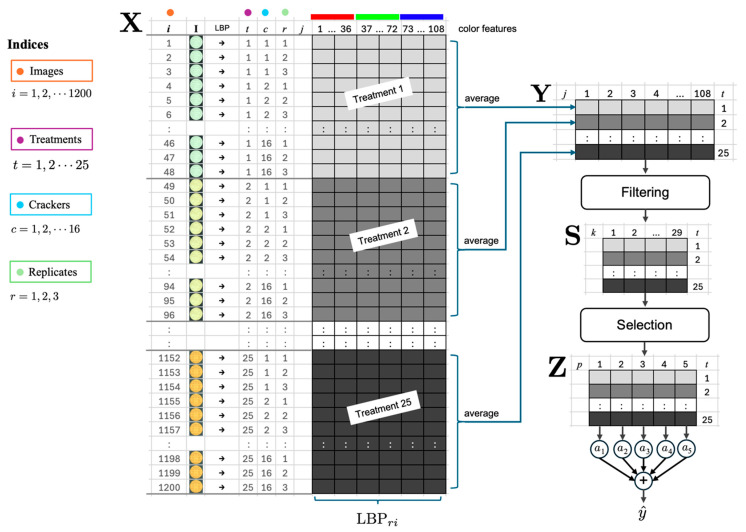
Feature extraction and processing diagram. X: color image matrix; Y: treatment-average matrix; S: filtered data matrix; Z: selected feature matrix; a_1_, a_2_, a_3_, a_4_, a_5_: linear coefficients. Red, green, and blue represent the RGB color components.

Finally, each column of our matrix X is linearly scaled between 0 and 1, for *j* = 1, …, 108. Linear normalization of features equalizes the influence of different features by scaling them uniformly, which enhances the performance and convergence of machine learning algorithms. Additionally, it helps maintain consistency during further data processing and visualization, ultimately contributing to better model generalization. The normalized matrix X is shown in Figure 3.

#### 2.4.5. Averaging of Features per Treatment

For each of the 25 treatments, there are 48 = 3 × 16 images (3 groups and 16 images per group), that correspond to the 48 measurements of the 108 features extracted in the 25 treatments were averaged (see Y in Figure 3), as follows:

(4) $$y_{t}=\;\frac{1}{48}\sum_{i=i_{0}+1}^{i_{0}+48}x_{i}\;\mathrm{with}i_{0}=48(t-1)\mathrm{and}t=1,\ldots ,25$$

To facilitate industrial batch-level monitoring, our experimental design captures the mean characteristics of each treatment rather than individual unit variance. We mathematically averaged visual features from 48 images per treatment to structurally align with the homogenized chemical ground truth. This aggregation is essential because it filters out localized visual anomalies on individual crackers, prevents overfitting, and ensures the model learns the true mean batch response to processing parameters. Furthermore, model robustness is maintained through the wide inter-treatment variability generated across the 25 distinct baking conditions.

#### 2.4.6. Filtering

From the 108 characteristics, those with a standard deviation of less than 0.15 in each treatment are selected. In this way, those features that are relatively constant in each treatment are maintained. In this step, 79 characteristics are removed, and only 29 remain, for *k* = 1, …, 29 matrix S in Figure 3.

#### 2.4.7. Output Normalization

Normalizing variables of varying scales is crucial as it ensures each feature contributes equally to the analysis. This prevents features with larger values from disproportionately influencing the calculations and distorting the outcomes. The physical variable (*AA* or *HMF*) with 25 measurements is scaled non-linearly using the square root and then linearly between 0 and 1 as follows:

(5) $$y_{a}=lin\left(\sqrt{AA}\right)$$

(6) $$y_{h}=lin\left(\sqrt{HMF}\right)$$

for *AA* and *HMF* respectively, where the function “*lin*(*x*)” linearly scales *x* between 0 and 1, i.e., (*x* − min(*x*))/(max(*x*) − min(*x*)).

#### 2.4.8. Feature Selection

Using the Sequential Forward Selection method [48] implemented in the Balu library [47], the m = 5 features (*z*_1_, *z*_2_, *z*_3_, *z*_4_, *z*_5_), that maximize the correlation between the output with a linear combination of the selected features are selected. Thus, we seek for the best five features of S, that maximizes:

(7) $$corr\left(y,\hat{y}\right)\to max$$

where:

(8) $$\hat{y}=\;a_{0}+\;a_{1}z_{1}+a_{2}z_{2}+a_{3}z_{3}+a_{4}z_{4}+a_{5}z_{5}$$

In our case, we repeat this method for *y* = *y_a_*, using (5) for the *AA*, and *y* = *y_h_*, using (6) for HMF.

To mitigate the risk of overfitting inherent in a dataset of 25 treatments, the dimensionality of the predictive model was strictly constrained. Before selection, the initial 108 LBP features were reduced to 29 by discarding those with a standard deviation below 0.15. Subsequently, the Sequential Forward Selection (SFS) algorithm was capped at exactly five features. This enforces a conservative 5-to-1 sample-to-feature ratio, which is optimal for linear models trained on small datasets. Furthermore, during the 25-fold ‘leave-one-treatment-out’ cross-validation, feature selection demonstrated robust stability across folds, confirming that the selected textural features consistently represent the variance in chemical contaminant formation rather than localized dataset noise.

#### 2.4.9. Extra Step

Given that HMF is considerably more straightforward and cost-effective to measure than *AA*, we explored the feasibility of estimating *y_a_* using *y_h_* alone, that is:

(9) $${\hat{y}}_{a}=a_{0}+\;a_{1}y_{h}$$

and in conjunction with the visual features extracted in previous steps, that is:

(10) $${\hat{y}}_{a}=a_{0}+\;{a_{1}z_{1}+a_{2}z_{2}+a_{3}z_{3}+a_{4}z_{4}+a_{5}z_{5}}_{1}$$

### 2.5. CATA (Check-All-That-Apply) Sensory Test

Three cracker baking treatments were selected for consumer preference analysis using the CATA test based on: (i) visual surface color evaluation, (ii) an AA content lower than the European Commission benchmark value of 400 µg/kg. The selected cracker samples were the ones baked at 180 °C, 190 °C, and 200 °C for 25 min to avoid excessive surface darkening, high AA levels and to ensure that the crunchiness attribute was present in all samples. A total of 101 consumers participated in this preference test.

The CATA test was conducted in the Food Processing Laboratory at the University of Chile, located within the Faculty of Chemical and Pharmaceutical Sciences. The samples were presented monadically and randomly on glass plates, each identified with a three-digit numerical code. Purified water was provided as a neutralization medium between samples, and consumers were instructed to take a few sips of water before tasting each sample. The sensory evaluations were carried out by students and staff from the University of Chile. The study was conducted in accordance with the tenets of the Declaration of Helsinki [49] and was approved by the Faculty of Chemical and Pharmaceutical Sciences, University of Chile Ethics Committee.

For the CATA test, descriptors (words or phrases) were presented according to previous studies by Vidal et al. [50] for crackers, with some modifications. Since this method is consumer-oriented, the determination of attributes was carried out using concepts simple enough for consumers to understand and apply. The selected attributes were: toasted color, light color, thin, thick, greasy sensation, dry, crunchy, soft, hard, brittle, salty, toasted flavor, bitter flavor, tasteless, aftertaste, would consume frequently, would not consume, would recommend to family or friends, with 1 indicating the attribute selected by the consumer and 0 indicating it was not selected. The test instructions also indicated that if there was any attribute they did not understand, they could ask for clarification.

The statistical analysis of the data from the CATA test was conducted using correspondence analysis for non-parametric data and Cochran’s Q test, utilizing XLSTAT Version 2014.5.03 software developed by Addinsoft.

## 3. Results and Discussion

### 3.1. Acrylamide and 5-Hydroxymethylfurfural Formation

**Figure 4:**
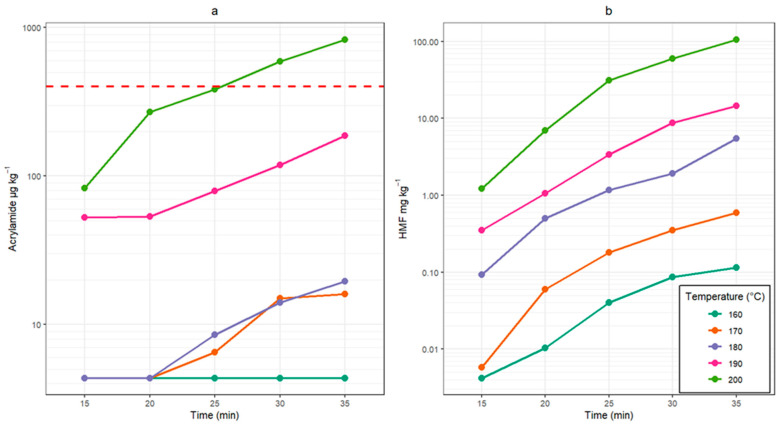
Log-linear plots of the formation of AA (**a**) and HMF (**b**) at different temperature–time baking conditions tested for cracker production. The red dashed line in (**a**) indicates the EU AA benchmark level for crackers (400 µg kg^−1^).

The formation of AA and HMF at different baking temperatures and times is shown in Figure 4. Both NFCs levels were dependent on the baking time and temperature. Interestingly, no significant AA formation was found at 160 °C regardless of baking time, and at 170 °C and 180 °C the AA levels started to increase from 25 min of baking onwards. Similar results were found by Hradecka et al. [51] in white flour crackers baked at 180 °C where AA levels were below the limit of quantification (<10 µg kg^−1^) for 25 min of baking time and increased at longer times, reaching mean values of 122 µg kg^−1^ at 35 min and 594 µg kg^−1^ at 45 min. At the higher temperatures (190 °C and 200 °C) AA formed at all baking times and it increased exponentially (Figure 4a). Only at 200 °C and with baking times of 30 and 35 min the AA levels exceeded the EU benchmark level of 400 µg kg^−1^. Flour composition largely influences the formation of AA, for instance flours with higher levels of sugars and asparagine such as whole grain wheat flour produced crackers with AA levels exceeding the benchmark value after baking at 180 °C and 35 min (517 µg kg^−1^, [51]). The average AA levels found in crackers were lower than the amounts found in other bakery products such as cookies (which include sugars in the formulation) baked at similar temperatures and times [52] highlighting the influence of free sugars in AA formation. In the case of HMF, it was detected at all baking temperatures and increased with baking times with the highest increase at 200 °C where it increased by roughly two orders of magnitude from 15 min (1.2 mg kg^−1^) to 35 min (105.4 mg kg^−1^) (Figure 4b). HMF is produced during baking by thermal decomposition and hydrolysis of sugars [53]. In the case of crackers, maltose and sucrose are the main sugars present in white wheat flour and thus are the main substrates for HMF formation [51].

**Figure 5:**
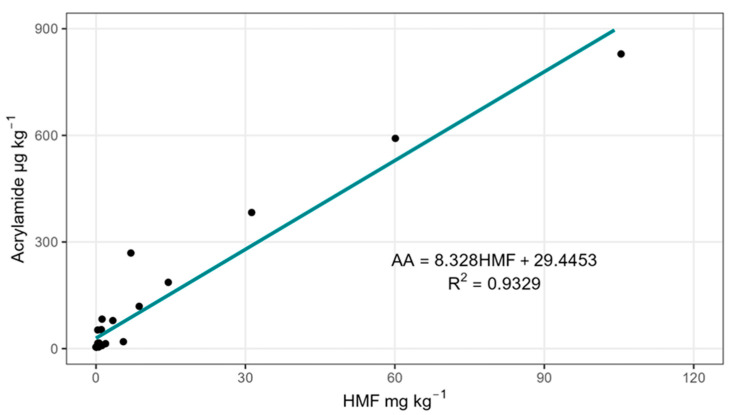
Correlation between AA and HMF experimental values in crackers.

Previous studies in both model systems and food matrices have shown that HMF could play a role as an AA intermediate in the Maillard reaction. In this study, HMF and AA concentrations were found to be highly linearly correlated (R^2^ = 0.9329, Figure 5). In fried potato chips, exponential (R^2^ = 0.85, [20]) and linear (R^2^ = 0.7591, [21]) relationships have been found between HMF and AA. It has been reported that HMF reacts with asparagine at a higher rate than glucose to form AA [54]. Therefore, both our results and previously published studies suggest that HMF levels could be a good predictor of the AA content in thermally treated foods (such as crackers). Nevertheless, further studies focusing on the formation mechanism of AA from HMF in real food matrices should be conducted.

### 3.2. Computer Vision

The process described in previous section is performed 25 times, for each treatment using the strategy of “leave-one-treatment-out”, that is a cross-validation methodology with 25 folds. The idea is to estimate the measurement of one treatment using the remaining 24 treatments. In this method, one treatment is left out (to leave it as a test), and the five characteristics are selected using the data from the remaining 24 treatments.

**Figure 6:**
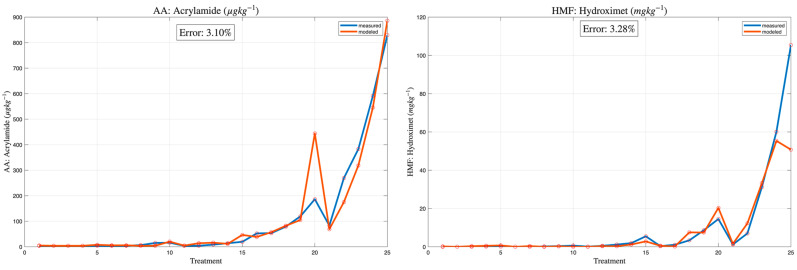
Comparison of measurements and estimations for AA and HMF using cross-validation from visual features.

Using Equation (8) we obtained estimations of the normalized AA and HMF, these values are then transformed to the original scale with the inverse of Equations (5) and (6) respectively. The results are given in Table 3 and Figure 6, where the average error is 3.10% for AA and 3.28% for HMF. In this case, the error has been calculated as the mean of the absolute difference between modeled and measured divided by the maximum of the measured values.

**Figure 7:**
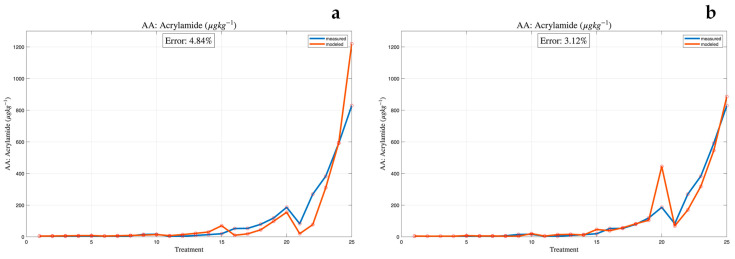
Estimation for AA with cross-validation using (**a**) HMF only and (**b**) HMF and visual features.

Finally, if we aim to estimate the variable AA from HMF, as outlined in step 9 of the preceding section, we utilized the same 25-group cross-validation method to obtain the following results: Estimating AA solely from HMF, as per Equation (9), results in a considerable higher error rate of 4.84% (refer to Figure 7a). Additionally, when AA is estimated using both HMF and visual characteristics, according to Equation (10), the error rate is reduced significantly to 3.12% (see Figure 7b). Notably, this error is slightly higher than 3.10%, the error rate achieved when the model does not incorporate the HMF variable.

Our experiments demonstrate that standardized image acquisition procedures, utilizing controlled lighting and consistent camera–object distances, enable the accurate estimation of AA and HMF levels directly from visual features extracted from cracker images. This finding is highly significant: by relying exclusively on computer vision, we can eliminate the need for time-consuming and costly analytic chemical measurements. Furthermore, because this vision-based approach bypasses expensive physical sampling entirely, it opens the door to implementing this system as a real-time monitoring instrument directly within the production chain. Regarding the estimation models, our results indicate that predicting AA solely from HMF is not advisable due to higher error rates. Interestingly, while combining HMF with visual features significantly reduces this error, the error rate (3.12%) remains slightly higher than when AA is calculated entirely from visual features alone (3.10%, as shown in Equation (10)), further underscoring the efficacy of a purely vision-based approach.

While the overall mean errors for the model are highly competitive, Table 3 highlights that absolute deviations increase under extreme baking conditions (e.g., 200 °C for extended times). This occurs because the relationship between surface browning and acrylamide is not perfectly linear across all stages of heating. At extreme temperatures, contaminant formation becomes highly exponential, while surface darkening begins to visually saturate the linear model to under- or over-predict the precise extreme value. However, this does not affect the system’s practical reliability. For extreme treatments, the model still confidently predicts values far exceeding the 400 µg kg^−1^ EU benchmark, successfully flagging the batch as a safety failure. Furthermore, sensory CATA data confirms that crackers baked at these extreme conditions are firmly rejected by consumers due to burnt flavor and color (See Section 3.3), rendering the precise quantification of these extreme outliers less critical for industrial application.

The 5 × 5 factorial design yields 25 distinct treatments; however, this represents a relatively small sample size for training predictive models, carrying a risk of overfitting. To ensure robust generalization, the methodology enforces strict dimensionality control. By restricting Sequential Forward Selection to exactly five variables, the model maintains a conservative 5:1 sample-to-feature ratio, ideal for linear regression. Furthermore, the input vector for each of the 25 treatments is highly stabilized, being derived from the average of 48 individual images, effectively filtering out visual noise. Combined with a 25-fold ‘leave-one-treatment-out’ cross-validation, this simple linear architecture ensures the model captures the underlying kinetics of browning rather than memorizing a limited dataset.

A detailed analysis of Table 3 reveals that the model’s accuracy varies depending on the thermal regime:

Standard Baking Conditions (160–180 °C): In treatments 1–15, which represent standard and consumer-acceptable baking parameters, the model demonstrates exceptionally high precision. The absolute differences between measured and modeled AA are negligible, often within a few µg kg^−1^ (e.g., Treatments 1–7). This precision in the lower-temperature range is critical, as it confirms that the CV system can reliably monitor normal industrial production without triggering false safety alarms.

Extreme Baking Conditions (190–200 °C): As processing temperatures reach 190 °C and 200 °C (Treatments 16–25), absolute errors increase noticeably. For instance, in Treatment 20 (190 °C, 35 min), the model significantly overestimates the AA content (modeled 444.3 vs. measured 186.5 µg kg^−1^), and in Treatment 22, it underestimates it (modeled 174.9 vs. measured 269.0 µg kg^−1^). These discrepancies occur because, under extreme thermal conditions, the kinetics of AA and HMF formation become highly exponential, while the visual browning of the cracker surface approaches saturation. Consequently, the strictly linear relationship between color/texture features and chemical concentration begins to decouple, leading to larger absolute deviations.

Despite higher absolute errors at these extreme temperatures, the system’s practical reliability remains intact. The primary objective of the CV model is to flag batches that exceed the EU benchmark of 400 µg kg^−1^. In extreme cases where safety is genuinely compromised (e.g., Treatments 24 and 25, measured at 591.5 and 829.0 µg kg^−1^, respectively), the model correctly predicts overwhelmingly high values (545.5 and 886.0 µg kg^−1^), successfully identifying the safety failure. Furthermore, as demonstrated by the sensory CATA results, crackers subjected to these extreme conditions are strongly rejected by consumers due to burnt flavor and texture, meaning they would be discarded for quality reasons regardless of precise analytical quantification.

While the computer vision methodology presented here is highly robust, it is important to note that the specific predictive model coefficients are matrix-dependent. Variations in dough formulation (e.g., different sugar or amino acid profiles) or industrial baking mechanics (e.g., tunnel ovens versus convection ovens) will alter the specific relationship between surface browning and contaminant formation. For real-world industrial applications, this CV framework is highly generalizable. Still, it would require a standard calibration phase (correlating initial visual features of the new formulation with baseline chemical measurements) to update the linear weights before autonomous, real-time monitoring can commence.

### 3.3. CATA Test

The responses of 101 consumers who reported consuming crackers at least once a month were considered for CATA test. Among them, 52% were female, 46% were male, and 2% did not declare their gender. Finally, 87% of these consumers were in the age range of 18–25-year-old, 10% were in the age range of 26–30-year-old, 2% were in the age range of 31–40-year-old, and 1% were 41-year-old or older. Therefore, the obtained results could only be extrapolated to young people (18–25-year-old) in similar conditions within a population.

**Figure 8:**
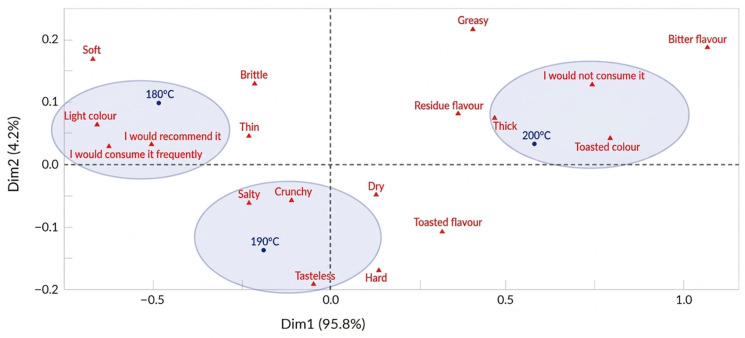
Sensory profiles of the three cracker samples (baked at 180 °C, 190 °C, and 200 °C for 25 min) based on correspondence analysis of the CATA test.

According to Figure 8, consumers preferred the crackers baked at 180 °C for 25 min, which also had the lowest levels of AA and HMF among the three samples sensorially tested (8.5 mg kg^−1^ and 1.2 mg kg^−1^, respectively). Crackers baked at 180 °C for 25 min were associated by consumers with being soft, brittle, thin, and light in color. Additionally, due to the lower baking temperature, their moisture loss was lower compared to the other two samples. This sample was also selected by consumers with the statements “I would recommend it” and “I would consume it frequently”. When the crackers were baked at 200 °C for 25 min, consumers perceived them as having a thick texture, a residual flavor, and a toasted color, and they indicated they would not consume them. This sample also contained the highest levels of AA and HMF among the three samples sensorially tested (383 μg kg^−1^ and 31.3 mg kg^−1^, respectively). Its AA content was very close to the benchmark level of 400 μg kg^−1^ for crackers. Finally, crackers baked at 190 °C for 25 min were described by consumers as salty, crunchy, and tasteless. As expected, their AA and HMF contents were intermediate between those of the samples baked at 180 °C and 200 °C (79 μg kg^−1^ and 3.4 mg kg^−1^, respectively).

In visual perception, the attribute color intensity (light color), and in the mouth, texture attributes (soft, brittle and thin) were decisive in the consumers preference (I would recommend it and I would consume it frequently), to choose the crackers baked at 180 °C for 25 min. These results agree with the studies on biscuits by Ervina [55], in addition to color intensity and textural attributes, sweetness and bitterness were considered in the hedonic and sensory characteristics of the crackers, using the CATA method as a descriptive analysis of consumers.

It is important to note that a closer relationship between computer vision and sensory analysis would require a more detailed study designed in such a way that sensory variables could be directly related to visual and chemical features. On the other hand, it is important to emphasize that sensory analysis encompasses a broad spectrum of perception, involving all the senses and how consumers perceive those sensorial attributes. Therefore, reducing sensory perception only to visual features may seem interesting from a market quality perspective; however, the consumer’s sensory perspective is the interaction between of all the senses and should be considered.

## 4. Conclusions

This work showed that HMF formation in salty crackers is highly correlated with acrylamide and thus could be used as a predictor for the levels of this harmful neo-contaminant. Furthermore, by applying a computer vision-based approach, AA content of crackers could be accurately predicted after its validation under standard industrial production conditions, reducing the need for conventional analytical techniques at an industrial scale. In conjunction with sensory analysis, a systematic approach to estimate both neo-formed contaminants and consumer preference could be developed which can greatly improve the development of new bakery products.

Our experiments demonstrate that under strictly standardized image acquisition procedures (utilizing controlled lighting and consistent camera–object distances) the extracted visual features allow for highly stable estimations of AA and HMF levels. While the predictive model demonstrated high statistical reliability across the 25 distinct baking treatments evaluated in this study, it is important to clarify that this algorithmic stability relies on the uniform optical environment. The hardware and environmental robustness of this technique (such as its performance using different camera sensors or under fluctuating ambient factory conditions) has not yet been evaluated and remains an important next step before industrial deployment. Transitioning this technique from a controlled laboratory prototype to a seamless industrial tool will require the development of integrated, commercially viable devices (similar to current inline colorimeters) that encapsulate the necessary controlled lighting, camera hardware, and predictive software.

This work demonstrates that computer vision can accurately predict AA and HMF content; however, it is not intended to eliminate the need for conventional analytical techniques completely. In an industrial environment, potential lighting variability and product heterogeneity must be managed by deploying the camera within a strictly controlled, enclosed illumination setup. Ultimately, this CV approach is proposed as a real-time, non-destructive screening tool to reduce reliance on routine, batch-by-batch chemical sampling drastically. Conventional analytical methods remain essential for baseline calibration, periodic auditing, and the validation of borderline batches, ensuring a robust framework that combines rapid AI estimation with necessary human validation.

## Figures and Tables

**Table 1:** Cracker dough formulation *.

Ingredients	Weight (g)
Wheat flour	100
Vegetal lard	7
Salt	2
Water	42

* Based on 100 g of wheat flour.

**Table 2:** Full factorial design for the different temperature–time baking.

Treatment *	Temperature (°C)	Time (min)
1	160	15
2	160	20
3	160	25
4	160	30
5	160	35
6	170	15
7	170	20
8	170	25
9	170	30
10	170	35
11	180	15
12	180	20
13	180	25
13	180	30
15	180	35
16	190	15
17	190	20
18	190	25
19	190	30
20	190	35
21	200	15
22	200	20
23	200	25
24	200	30
25	200	35

* Twenty-five combinations of temperature and baking time treatments were tested in random order. Both the baking and image acquisition processes were conducted in triplicate. Sixteen pieces were baked under each condition, resulting in 48 crackers per treatment and a total of 1200 photographs of the manufactured crackers.

**Table 3:** Measured and modeled values for AA and HMF.

	AA: Acrylamide (µg kg^−1^)			HMF: Hydroxymethylfurfural (mg kg^−1^)		
Treatment	Measured	Modeled	Error	Measured	Modeled	Error
1	4.0	5.1	1.1	0.00	0.18	0.2
2	4.0	4.0	0.0	0.01	0.00	0.0
3	4.0	4.0	0.0	0.04	0.32	0.3
4	4.0	4.0	0.0	0.09	0.53	0.4
5	4.0	7.8	3.8	0.11	0.71	0.6
6	4.0	5.7	1.7	0.01	0.00	0.0
7	4.0	5.8	1.8	0.06	0.37	0.3
8	6.5	4.0	2.5	0.18	0.00	0.2
9	15.0	4.2	10.8	0.35	0.25	0.1
10	16.0	20.2	4.2	0.59	0.00	0.6
11	4.0	4.7	0.7	0.09	0.00	0.1
12	4.0	13.9	9.9	0.50	0.34	0.2
13	8.5	16.0	7.5	1.17	0.30	0.9
14	14.0	11.6	2.4	1.92	1.20	0.7
15	19.5	46.5	27.0	5.49	2.81	2.7
16	52.5	38.6	13.9	0.35	0.53	0.2
17	53.5	57.1	3.6	1.06	0.20	0.9
18	79.0	82.3	3.3	3.38	7.53	4.1
19	119.0	104.9	14.1	8.67	7.45	1.2
20	186.5	444.3	257.8	14.51	20.33	5.8
21	83.0	68.8	14.2	1.22	1.55	0.3
22	269.0	174.9	94.1	7.00	12.24	5.2
23	383.0	318.5	64.5	31.25	33.27	2.0
24	591.5	545.5	46.0	60.10	55.34	4.8
25	829.0	886.0	57.0	105.42	50.75	54.7
		MEAN =	3.10%		MEAN =	3.28%

## Data Availability

The original contributions presented in this study are included in the article/Supplementary Material. Further inquiries can be directed to the corresponding authors. The computational code for image processing and the 1200 digital images acquired and analyzed in this study can be accessed at (URL will be available once the manuscript is accepted for publication).

## Supplementary Materials

The following supporting information can be downloaded at: https://www.mdpi.com/article/10.3390/foods15112011/s1. The main analytical validation parameters for AA and HMF chromatographic methods is provided.

## Abbreviations

The following abbreviations are used in this manuscript:

AA	Acrylamide
CATA	Check-All-That-Apply
CV	Computer Vision
HMF	5-Hydroxymethylfurfural
MR	Maillard reaction
NFC	Neo-formed Contaminant
