# Supplementary material for: Estimating Acrylamide and 5-Hydroxymethylfurfural Levels in Crackers Using Computer Vision: Effects on Consumer Acceptance
Source: Foods. 2026 Jun 4;15(11):2011. doi: 10.3390/foods15112011 (PMC13257408; doi:10.3390/foods15112011)
Supplement: Supplementary file 1 [file foods-15-02011-s001.zip › foods-4322757-supplementary.pdf]

Supplementary material

Analytical validation parameters

1.1. Acrylamide

1.1.1. Linearity

Linear range: 0.025-1 µg mL^{-1}

$R^2 = 0.9996$

LOD (S/N = 3) = 0.003 µg mL^{-1}

LOQ (S/N = 10) = 0.009 µg mL^{-1}

1.1.2. Precision

Repeatability: SD = 0.052 µg mL^{-1}, RSD = 9.11% (n = 7)

1.1.3. Accuracy

Recovery = 101.76%

1.2. 5-Hydroxymethyl furfural

1.2.1. Linearity

Linear range: 0.03-3 µg mL^{-1}

$R^2 = 0.9999$

LOD (S/N = 3) = 0.003 µg mL^{-1}

LOQ (S/N = 10) = 0.011 µg mL^{-1}

1.2.2. Precision

Repeatability: SD = 0.017 µg mL^{-1}, RSD = 1.89% (n = 7)

1.2.3. Accuracy

Recovery = 83.99%
